# Remediation potential of mining, agro-industrial, and urban wastes against acid mine drainage

**DOI:** 10.1038/s41598-023-39266-4

**Published:** 2023-07-26

**Authors:** Antonio Aguilar-Garrido, Mario Paniagua-López, Manuel Sierra-Aragón, Francisco Javier Martínez Garzón, Francisco José Martín-Peinado

**Affiliations:** Departamento de Edafología y Química Agrícola, Facultad de Ciencias, Universidad de Granada, Avda. de Fuente Nueva S/N, 18071 Granada, Spain

**Keywords:** Environmental sciences, Environmental impact

## Abstract

Acid mine drainage (AMD) poses serious consequences for human health and ecosystems. Novel strategies for its treatment involve the use of wastes. This paper evaluates the remediation potential of wastes from urban, mining and agro-industrial activities to address acidity and high concentrations of potentially toxic elements (PTE) in AMD. Samples of these waste products were spiked with an artificially prepared AMD, then pH, electrical conductivity (EC), and PTE concentrations in the leachates were measured. The artificial AMD obtained through oxidation of Aznalcóllar’s tailing showed an ultra-acid character (pH − 2.89 ± 0.03) and extreme high electrical conductivity (EC − 3.76 ± 0.14 dS m^−1^). Moreover, most PTE were above maximum regulatory levels in natural and irrigation waters. Wastes studied had a very high acid neutralising capacity, as well as a strong capacity to immobilise PTE. Inorganic wastes, together with vermicompost from pruning, reduced most PTE concentrations by over 95%, while organic wastes retained between 50 and 95%. Thus, a wide range of urban, mining, and agro-industrial wastes have a high potential to be used in the treatment of AMD. This study provides valuable input for the development of new eco-technologies based on the combination of wastes (eg. Technosols, permeable reactive barriers) to remediate degraded environments.

## Introduction

Mining is a crucial industry worldwide because of its economic and social relevance, as it supplies a large number of important resources. In recent decades, the number of operating mines has increased significantly due to the need for strategic elements (e.g., critical metals, rare earth elements, platinum-group elements, technology-critical elements), raising health and environmental concerns^1–3^. Sulfides are the main supplier of a broad range of metal(loid)s, that can be considered as potentially toxic elements (PTE), and their exploitation is one of the world’s most important mining activities^4^. The exposure of these sulfides (pyrite ore [FeS_2_] mainly), or their wastes, to oxidising and rainfall conditions leads to the generation of acid mine drainage (AMD), which is commonly associated with serious environmental problems worldwide^5^; particularly, in abandoned or active mines without legal concessions (i.e., extraction areas without environmental management of drainage and wastes). Acid mine drainage is problematic because of its scale, both in space and time, as it can affect both mining areas and their surroundings over large kilometres for decades or centuries^6^. Furthermore, AMD poses serious consequences for human health (e.g., nervous system damage, cancers, mental retardation in children) and the ecosystems (e.g., groundwater pollution, phytotoxicity and inhibition of photosynthesis, fish mortality)^7–10^. A good example of this concern can be found in the Iberian Pyrite Belt (Southeast Portugal and Southwest Spain), one of the largest massive sulfide reserves in the world, where large-scale mining activities date back to the nineteenth century and earliest activities to the 3^rd^ millennium BC^11^. In this region, AMD is a legacy of abandoned mines and associated tailing dumps, including enormous sulfide-bearing waste rock piles, tailings, and flooded pits, as well as waste produced by operating mines^12,13^. Thus, this region constitutes a potential source of AMD pollution (Fig. S1) and is representative of other sulfide mines located all around the world.The discharge of untreated AMD exerts negative effects on environment. In aquatic ecosystems, it is responsible for the entry of PTE into these media, the alteration of water chemistry and nutrient cycles, the decrease in the amount of oxygen available to organisms, and the precipitation of metals (Fe and Al hydroxides), among others. In general, water quality is affected, causing direct toxicity to organisms and rendering it unsuitable for domestic, agricultural and industrial uses^9,14,15^. In terrestrial ecosystems, the untreated discharge of AMD can lead to soil pollution, and consequently, accelerates biodiversity loss and soil degradation^9^. Moreover, AMD generated both in active and abandoned mining areas, can have several health impacts on environment and living organisms (including humans) by polluting surface water, groundwater and agricultural soils^8^.

Many technological solutions exist for the treatment of AMD involving chemical, physical and/or biological processes (e.g., oxidation, (bio)reduction, (bio)sorption, ion exchange, complexation, precipitation, dilution, alkaline generation)^5,8,9,16,17^. But these techniques are often costly (even unaffordable) and limited in field conditions and over time, even compromising the economic viability of entire mining projects, as their development demands relatively large capital investment on material handling, equipment and/or maintenance^5,16,18,19^. Thus, it is necessary to advance AMD remediation strategies that lead to improved, cost-effective, and environmentally friendly methods^8^. Recently, promising methods have focused on the use of low-cost amendments; for example, employing wastes from several human activities, to tackle the negative impacts of AMD and connect with the circular economy strategy^20–22^. In this sense, some research has explored the use of materials at the end-of-life-cycle from different sectors to control and treat AMD. For instance, wastes from steelmaking processes (slag materials) and gas treatment at a thermal power plant (fly ash and gypsum) had removed inorganic PTE (As, Hg, Pb, Zn, Cd, Cu and Ni) from AMD at the abandoned “La Soterraña” mercury mine (Asturias, Spain)^23^. Another example was the use of alkaline waste material from an alumina refining industry (“Bayer liquor” and precipitates formed by the seawater neutralization of this “Bayer liquor”) as an alternative to neutralise AMD from the Mount Morgan mine (Queensland, Australia), since they significantly buffer the acid pH and reduce Al, Cu, Fe, Zn and Ni levels^18^.

The unsustainable amount of waste generated today is also a main concern. For example, in 2018, a total of 2377 M tonnes of waste was generated in EU by all economic activities and households, of which, mining and quarrying activities, together with wastewater treatment, agriculture, forestry and fishing, and households contributed almost 46%^24^. In 2022, the world’s cities were estimated to generate 2240 M tonnes of municipal solid waste (MSW)^25^, with differences between geographical areas (in kg yr^−1^ ca^−1^; 800 in USA^26^, 657 in Australia^27^, 505 in EU^28^, 368 in Brazil^29^, 277 in China^30^, and 168 in India^31^). The mining waste amounts are even higher in mass than those of MSW. The estimated global generation of solid wastes from mineral and metal production is over 100,000 M tonnes per year^22^.In Europe, 636 M tonnes of mining and quarrying wastes were generated in 2018 (25% of all waste produced in the EU)^24^. Agro-industry, also essential in the primary sector, generates 140,000 M tonnes of waste each year: mainly maize stalks, straw, sugarcane leavings, bagasse, manure from cattle, poultry, and pigs, forestry residues, and garden pruning^32^. The main global crops (wheat, maize, rice, soybean, barley, rapeseed, sugarcane and sugar beet) produced generates almost 3300 M tonnes waste, where China, USA, India and Europe are among the biggest producers (with 716, 682, 605, and 580 M tonnes, respectively)^33^. Other example comes from our study area, Andalusia (Southeast Spain), where the production of olive oil creates 6 M tonnes of waste every year^34^. So, there is an urgent need for waste policies to move towards approaches that contribute to the circular economy by extracting high quality resources from waste as far as possible.

Most of the literature published to date on eco-technologies to remediate AMD based on the use of end-of-life materials focused on industrial and mining waste but little attention has been paid to the use of other materials coming from different activities such as agro-industrial or urban wastes. Here, we evaluate the remediation potential of a wide range of inorganic and organic wastes coming from a large variety of human activities to cope against acidity and PTE concentrations of AMD. In particular, the aim of this study is to evaluate the acid neutralisation capacity and the removal effectiveness of PTE present in an acid mine drainage (AMD) of ten different inorganic and organic waste materials, involving the main waste-generating activities (urban, mining and agro-industrial activities), to contribute to the implementation of new eco-technologies for the treatment of AMD in a circular economy scenario.

## Material and methods

### Waste characterization

**Figure 1:**
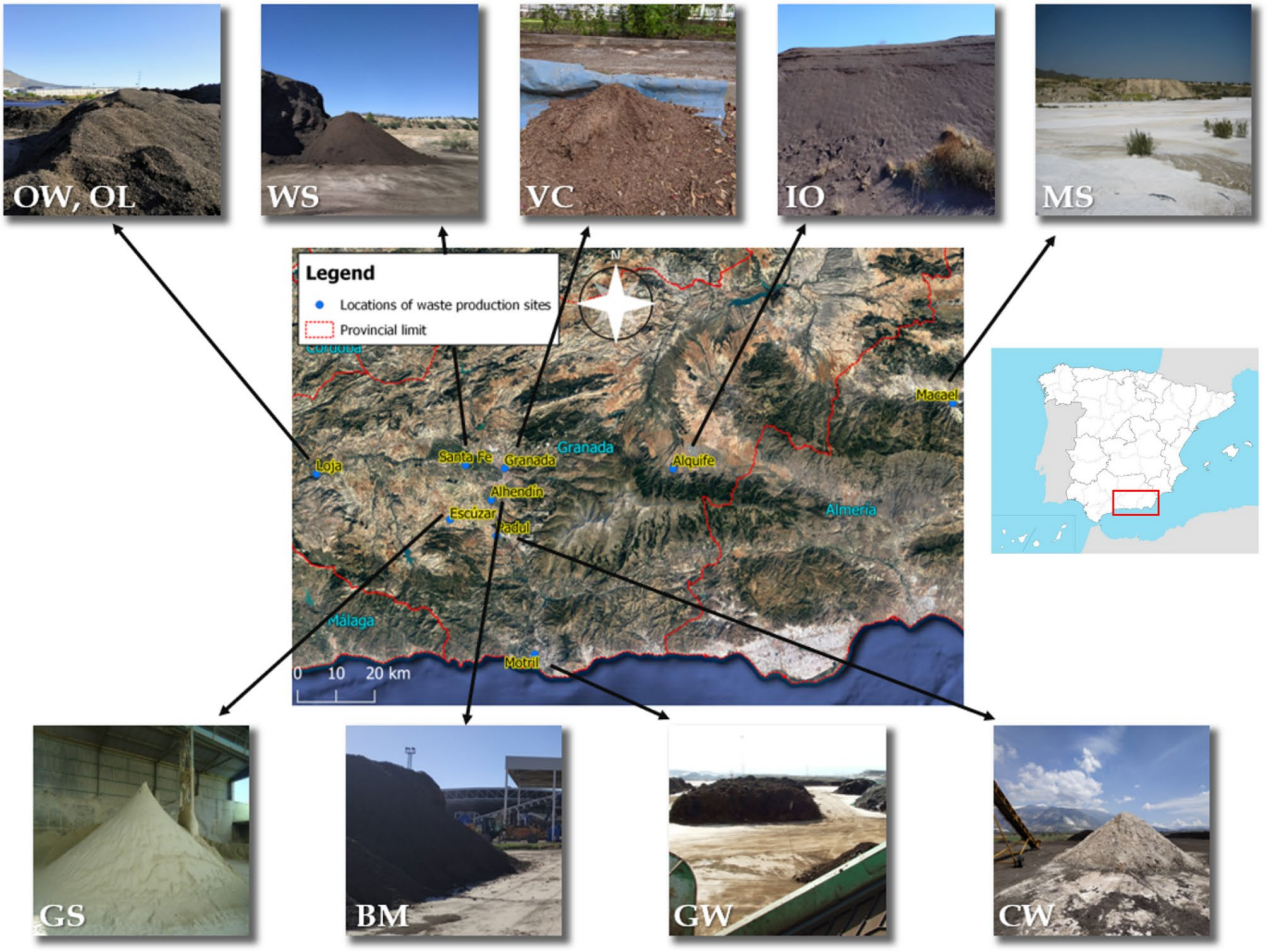
Location of waste production sites in Southeast Spain. This satellite imagery was generated using QGIS 3.20 Odense software (https://www.qgis.org/es/site/index.html) and orthophotography provided by OpenStreetMap (https://www.openstreetmap.org/#map=6/40.007/-2.488).

In this study, a total of 10 waste materials (4 inorganics and 6 organics) available in the case study region (Southeast Spain) have been selected, since the proximity of these materials is a key factor in the cost effectiveness of the remediation treatments applied (Fig. 1). These waste materials come from common activities all over the world in urban, mining and agro-industrial environments and are thus assumed to be readily available in other regions affected by AMD. Inorganic wastes are of mining origin and included the following: (i) dry sludges rich in iron oxyhydroxides (IO), (ii) dry sludges from the cutting and polishing of marble (MS), (iii) carbonated wastes from a peat bog mining (CW), and (iv) gypsum mining spoils (GS). Organic wastes come from both urban and agro-industrial activities. Those of urban origin are: (i) composted sewage sludges (WS), (ii) bio-stabilised material from a municipal solid waste plant (BM), and iii) vermicompost produced from pruning and gardening (VC); those of agro-industrial origin are: (i) and (ii) two different composted solid olive-mill by-products (OW: one irrigated with drinking water, OL: another irrigated with a liquid waste product of the olive-mill), and iii) compost of agricultural greenhouse plant waste (GW).

Main physicochemical parameters of the waste materials were analysed in triplicate in ground and homogenised samples: pH in a water extract (1:2.5 m:V) with a 914 pH/conductivity-meter Metrohm (Metrohm AG, Herisau, Switzerland); electrical conductivity (EC) in a water extract (1:5 m:V) using an Eutech CON700 conductivity-meter (Oakton Instruments, Vernon-Hills, IL, USA); organic carbon content (OC) by wet oxidation^35^; calcium carbonate content (CaCO_3_) by volumetric gases using a modified Bernard calcimeter^36^; exchangeable bases (Ca, Mg, Na, K) were determined after saturation with ammonium acetate (pH 7) and cation exchange capacity (CEC) after saturation with sodium acetate (pH 8.2) according to the extraction method^37^ and measured by atomic absorption spectroscopy using a VARIAN SpectrAA 220FS (Varian Associates, Palo Alto, California, USA); total nitrogen (N_T_) and carbon (C_T_) were analysed by dry combustion using an elemental analyser LECO TruSpec CN (LECO Corporation, St. Joshep, MI, USA); and assimilable phosphorus concentration (P_A_) by extraction with 0.5 M NaHCO_2_ (pH  8.5) and subsequent evaluation by colorimetry in Spectronic Helios γ UV–vis spectrophotometer (Thermo Fisher Scientific Inc., Waltham, MA, USA) using a solution of ammonium molybdate and ascorbic acid^38^. Furthermore, heterotrophic respiration was measured by determining the CO_2_ flux from waste material with a microbiological analyser μ-Trac 4200 SY-LAB model (SY-LAB Geräte GmbH, Neupurkersdorf, Austria) according to ISO 17,155^39^ and the results were expressed as the basal respiration rate (BR) in μg CO_2_ day^−1^ kg soil^−1^.

Total concentrations of PTE (As, Cd, Co, Cr, Cu, Fe, Ni, Pb, Sb, V, and Zn) were analysed in triplicate by inductively coupled plasma optical emission spectrometry (ICP-OES) in a spectrometer PerkinElmer Avio 500 (PerkinElmer, Inc., Waltham, MA, USA) after acid digestion (HNO_3_ + HF 3:1 V:V for inorganic wastes and HNO_3_ + H_2_0_2_ 1:1 V:V for organic wastes) in a Mars XP1500 Plus microwave (CEM Corporation, Matthews, CN, USA). The precision and accuracy of this method were assessed by measurement (three replicates) of a certified reference material (CRM BCR–482 EC-JRC-IRMM, Geel, Belgium). For all elements of interest, measured values were within the prediction interval of the certified value.

### Preparation of acid mine drainage

An artificial AMD was prepared in the laboratory following a method-based on the oxidation of pyritic tailings with hydrogen peroxide (H_2_O_2_)^40^. In detail, this pollutant solution used as AMD was prepared by progressive addition of 1 L H_2_O_2_ (33%) + 1 L H_2_O to 42.85 g of pyrite tailing and after three days, the solution was extracted by discarding the precipitated sediment, and then pH and EC (2.89 and 3.76 dS m^−1^, respectively) were measured. The pyrite tailing used comes from the Aznalcóllar mine (Seville, Spain), and belongs to the 0.9 × 10^6^ m^3^ of toxic tailings discharged into the Agrio and Guadiamar river basin, in one of the biggest mining accidents in Europe, the Aznalcóllar’s environmental disaster in 1998^41–43^. The PTE concentrations in the toxic tailings immediately after the accident (Table S1) were measured in previous studies^42,44^.

### Acid mine drainage treatment using waste materials

All waste materials were spiked with the acid mine drainage (AMD) prepared from the oxidation of pyritic tailings. This experience was made by the addition of 50 mL of AMD to 10 g of each waste material in triplicate to check the first impact of the AMD on different waste materials. Afterward, they were stirred for 24 h and filtered (Filter-Lab n°1250, pore size: 10–13 µm), separating the waste material (solid phase) from the leachate (liquid phase). In the leachate, which is the AMD treated, pH_(L)_ and EC_(L)_ were measured with a pH/conductivity-meter 914 Metrohm (Metrohm AG, Herisau, Switzerland) and an Eutech CON700 conductivity-meter (Oakton Instruments, Vernon-Hills, IL, Waltham, MA, USA), respectively, and PTE concentrations in solution were determined by inductively coupled plasma optical mass spectrometry (ICP-MS) in a spectrometer PerkinElmer NexION 300D (PerkinElmer, Inc., Waltham, MA, USA). The precision and accuracy of this method were assessed by measurement (three replicates) of a certified reference material (CRM BCR–482 EC-JRC-IRMM, Geel, Belgium). For all elements of interest, measured values were within the prediction interval of the certified value.

### Statistical analysis

A preliminary analysis of descriptive statistics was made. Non-parametric Kruskal–Wallis and Dunn tests (*p* < 0.05) for the analysis of mean comparison in the waste materials characterisation were chosen due to the sample size^45^. To analyse the results of the treatment of AMD by wastes, normality was checked with the Shapiro–Wilk test and homoscedasticity with the Levene test. As none of these conditions were met, even after transforming the variables, non-parametric Kruskal–Wallis and Dunn tests (*p* < 0.05) for multiple comparisons were applied. Furthermore, to analyse the influence of waste properties on their capacity of acid neutralisation and removal of PTE in polluted waters, significant bivariate Spearman’s correlations were also performed. All analyses were made with a confidence level of 95% by using RStudio software (RStudio Inc., 250 Northern Ave, Boston).

### Ethical approval

This study did not use any kind of human participants or human data, which require any kind of ethical approval or consent to participate.

### Consent to publish

Our study did not use any kind of individual data such as video and images.

## Results

### Characterisation of waste materials and acid mine drainage

All inorganic wastes were characterised by neutral to moderately alkaline pH (7.3–8.3), low organic carbon content (< 1.3% OC), low total nitrogen concentration (< 0.1% N_T_), and moderate to low cation-exchange capacity (CEC < 15 cmol^+^ kg^−1^) (Table 1). However, they were different in terms of other properties. Dry sludge rich in iron oxyhydroxides (IO) was dominated by iron (Fe_T_ ~ 87%), had moderate to low carbonation (~ 13% CaCO_3_) and very low EC (< 0.04 dS m^−1^). Dry marble sludge (MS) and carbonated waste (CW) had low values in total iron (< 0.3%), very high CaCO_3_ content (> 90%) and very high EC (> 1 dS m^−1^). Gypsum spoil (GS) had moderate to low values in total iron (~ 1%), moderately high CaCO_3_ content (~ 23%) and very high EC (> 2.9 dS m^−1^). The only inorganic waste that showed an assimilable phosphorus content (P_A_ ~ 470 mg kg^-1^) above detection limits was CW.

**Table 1:** Main properties of all waste materials (IO, MS, CW, GS, WS, BM, VC, OW, OL, GW).

Properties														
Waste	pH _(H2O) 1:2.5_	EC _1:5_ (dS m^-1^)	OC (%)	CaCO_3_ (%)	CEC (cmol^+^ kg^−1^)	Ca^2+^ (cmol^+^ kg^−1^)	Mg^2+^ (cmol^+^ kg^−1^)	Na^+^ (cmol^+^ kg^−1^)	K^+^ (cmol^+^ kg^−1^)	N_T_ (%)	C_T_ (%)	Fe_T_ (%)	P_A_ (mg kg^−1^)	BR (µg CO_2_ day^−1^ kg^−1^)
IO	7.27 ± 0.08 b	0.04 ± 0.01 a	n.d	13.08 ± 0.22 b	6.34 ± 0.38 a	4.44 ± 0.39 a	n.d	1.11 ± 0.03 ab	0.79 ± 0.01 a	n.d	1.55 ± 0.01 a	86.59 ± 0.88 b	n.d	45.91 ± 4.42 a
MS	8.27 ± 0.13 e	1.13 ± 0.08 ab	0.16 ± 0.11 a	99.99 ± 0.01 f.	9.34 ± 4.76 ab	5.59 ± 4.60 a	1.44 ± 0.27 a	1.48 ± 0.13 b	0.84 ± 0.02 a	n.d	11.81 ± 0.05 b	0.17 ± 0.01 a	n.d	29.46 ± 0.20 a
CW	7.83 ± 0.04 d	2.81 ± 0.03 bc	1.34 ± 0.03 a	93.12 ± 0.72 e	14.70 ± 0.50 b	2.75 ± 0.80 a	9.92 ± 0.67 cd	1.15 ± 0.04 ab	0.88 ± 0.01 a	0.07 ± 0.02 a	12.51 ± 0.03 b	0.28 ± 0.00 a	470.83 ± 6.16 e	124.05 ± 66.27 b
GS	7.53 ± 0.17 c	2.91 ± 0.05 bc	0.18 ± 0.03 a	23.84 ± 0.57 cd	8.48 ± 1.52 ab	7.18 ± 1.54 ab	0.88 ± 0.13 a	0.0030 ± 0.0004 a	0.39 ± 0.04 a	n.d	3.04 ± 0.03 a	1.01 ± 0.02 a	n.d	54.77 ± 26.30 a
WS	7.16 ± 0.02 b	10.13 ± 0.69 e	21.95 ± 2.91 bc	10.77 ± 0.92 ab	55.11 ± 5.81 d	24.94 ± 1.07 d	9.02 ± 0.71 c	5.67 ± 0.31 c	6.35 ± 0.19 b	3.13 ± 0.09 e	23.37 ± 0.35 c	2.01 ± 0.09 a	401.53 ± 14.07 cd	13.95 ± 1.73 a
BM	6.48 ± 0.01 a	12.37 ± 0.38 f.	28.23 ± 0.80 d	7.74 ± 0.64 a	40.49 ± 0.83 c	3.81 ± 2.28 a	4.51 ± 1.47 b	18.85 ± 1.16 f.	13.32 ± 0.83 c	1.55 ± 0.05 cd	29.45 ± 0.19 cd	0.47 ± 0.09 a	134.21 ± 6.99 a	40.75 ± 2.87 a
VC	7.35 ± 0.08 bc	0.39 ± 0.07 a	10.50 ± 4.08 b	24.87 ± 1.30 d	35.83 ± 2.16 c	23.25 ± 2.00 d	8.36 ± 0.24 c	1.20 ± 0.02 ab	3.02 ± 0.04 ab	0.64 ± 0.02 b	12.44 ± 0.18 b	0.98 ± 0.13 a	226.94 ± 37.65 ab	82.96 ± 9.36 ab
OW	8.57 ± 0.06 f.	2.57 ± 0.90 bc	23.28 ± 5.34 bc	22.62 ± 1.45 cd	80.90 ± 2.28 e	15.91 ± 1.73 c	7.57 ± 1.30 c	6.02 ± 0.11 c	51.41 ± 0.77 d	1.74 ± 0.02 d	25.94 ± 0.24 cd	0.65 ± 0.00 a	263.89 ± 32.58 b	47.75 ± 2.79 a
OL	8.63 ± 0.03 f.	3.67 ± 1.86 c	28.08 ± 1.76 d	20.96 ± 1.25 c	90.72 ± 3.57 f.	6.54 ± 2.00 a	2.22 ± 0.13 ab	8.81 ± 0.03 d	73.15 ± 1.75 e	1.63 ± 0.28 cd	30.60 ± 6.90 d	0.62 ± 0.00 a	318.70 ± 98.25 bc	70.70 ± 32.73 ab
GW	9.52 ± 0.03 g	7.39 ± 0.59 d	14.17 ± 7.12 b	12.25 ± 1.89 b	43.48 ± 0.60 c	12.85 ± 0.82 bc	11.80 ± 1.54 d	12.80 ± 0.50 e	6.03 ± 2.70 b	1.36 ± 0.03 c	15.64 ± 0.22 b	0.64 ± 0.08 a	402.73 ± 18.64 cd	49.88 ± 2.76 a

IO – Dry sludge rich in iron oxyhydroxides, MS—Dry marble sludge, CW—Carbonated waste of a peat exploitation, GS—Gypsum mining spoil, WS—Composted sewage sludge, BM—Bio-stabilised material of municipal solid wastes, VC—Vermicompost from pruning and gardening, OW—Composted solid olive-mill by-product irrigated with drinking water, OL—Composted solid olive-mill by-product irrigated with leachates of the olive-mill, GW—Composted greenhouse plant waste, EC—Electrical conductivity, OC—Organic carbon content, CaCO_3_—Calcium carbonate content, CEC—Cation exchange capacity, N_T_/C_T_/Fe_T_—Total concentrations of N, C, and Fe, P_A_—Assimilable phosphorus, BR—Basal respiration rate, n.d.—not detected. Letters represent significant differences among waste materials (Kruskal–Wallis and Dunn tests, *p* < 0.05).

Organic wastes showed significant differences in relation to the inorganic ones, mainly by the higher content in OC, CEC, exchangeable bases, total N, and available P (Table 1). Otherwise, differences between the organic wastes were also important. Organic carbon ranged between 10.5% in vermicompost from gardening (VC) and 28% in bio-stabilised material of municipal solid waste (BM) and composted solid olive-mill irrigated with olive leachate (OL); CEC varied between 36 cmol^+^ kg^−1^ in VC and 91 cmol^+^ kg^−1^ in OL; N_T_ was between 0.6% in VC and 3.1% in composted sewage sludge (WS); and P_A_ ranged between 134 mg kg^−1^ in BM and 403 mg kg^-1^ in compost of agricultural greenhouse (GW). For the other properties, no significant differences were observed with respect to the inorganic wastes, although among the organic wastes there were. In this way, pH ranged from 6.5 in BM and 9.5 in GW; EC was low for VC (< 0.4 dS m^−1^), very high for composted solid olive-mill irrigated with water (OW) and OL (2–4 dS m^−1^), and extremely high (> 7 dS m^-1^) for the rest; and CaCO_3_ was also detected in all cases, ranging from 7.7% in BM to 24.9% in VC. Basal respiration (BR) presented a wide range of values without significant differences between inorganic and organic wastes, with maximum of 124 µg CO_2_ day^−1^ kg^−1^ in CW and minimum of 14 in WS µg CO_2_ day^−1^ kg^−1^.

Total concentrations of PTE showed significant differences among wastes (Table 2). However, in general, between organic and inorganic wastes there were no clear differences, although the concentrations of Cr, Cu, Ni, and Zn were usually higher in organic wastes than in inorganic ones. Within the inorganics, IO and GS had the highest concentrations of most PTE, especially IO with concentrations of As, Pb, and Sb close to 24, 29, and 21 mg kg^−1^, respectively. Otherwise, MS presented very low concentrations for As, Pb, V, and Zn; while CW showed very low concentrations for Co, Cr, Ni, Sb, and V. The organic wastes presented low concentrations of As, Cd, Co, and Sb, with values below 5.3, 2, 5, and 0.4 mg kg^−1^, respectively. Lead showed differences between wastes, ranging between 2.4 mg kg^−1^ in OW and 52.6 mg kg^−1^ in BM; while V ranged between 12 mg kg^−1^ in BM and 27 mg kg^−1^ in WS. The elements with higher concentrations in relation to inorganic wastes also presented significant differences between organics; Cr ranged between 16 mg kg^−1^ in OW and 45 mg kg^−1^ in BM; Cu varied between 25 mg kg^−1^ in VC and 365 mg kg^−1^ in GW; Ni was between 9.7 mg kg^−1^ in OW and 21.5 mg kg^−1^ in BM; and Zn oscillated between 49 mg kg^−1^ in OW and 517 mg kg^−1^ in WS.

**Table 2:** Total concentrations of potentially toxic elements (PTE) in waste materials (IO, MS, CW, GS, WS, BM, VC, OW, OL, GW) expressed in mg kg^-1^.

Waste	As	Cd	Co	Cr	Cu	Ni	Pb	Sb	V	Zn
IO	23.92 ± 1.98 d	0.03 ± 0.02 ab	3.82 ± 0.52 cd	6.18 ± 0.56 ab	5.80 ± 0.28 a	6.96 ± 0.61abc	28.56 ± 2.94 c	20.85 ± 0.90 c	43.10 ± 2.88 d	25.86 ± 1.68 a
MS	0.77 ± 0.17 a	0.15 ± 0.02 abc	1.21 ± 0.04 ab	5.35 ± 0.18 a	4.01 ± 0.03 a	2.94 ± 0.09 ab	2.44 ± 0.35 a	0.33 ± 0.09 a	5.39 ± 0.31 ab	5.45 ± 0.80 a
CW	1.51 ± 0.37 a	0.26 ± 0.04 abc	0.15 ± 0.05 a	2.31 ± 0.47 a	13.78 ± 2.08 a	0.68 ± 0.37 a	9.01 ± 1.15 b	0.13 ± 0.33 a	2.58 ± 0.44 a	22.50 ± 1.89 a
GS	4.14 ± 0.25 bc	0.04 ± 0.05 ab	8.22 ± 1.84 e	11.46 ± 2.57 ab	11.27 ± 0.71 a	14.92 ± 3.38 de	3.23 ± 1.34 a	2.61 ± 0.48 b	25.64 ± 0.93 c	23.47 ± 5.19 a
WS	5.26 ± 1.76 c	0.72 ± 0.18 bc	3.88 ± 0.71 cd	33.61 ± 9.35 c	211.83 ± 9.52 c	20.75 ± 2.07 e	39.79 ± 5.15 d	n.d	26.79 ± 3.00 c	517.19 ± 67.54 d
BM	1.80 ± 0.17 ab	1.88 ± 0.37 d	2.37 ± 0.35 bc	45.07 ± 8.12 d	135.13 ± 24.11b	21.51 ± 4.52 e	52.61 ± 9.55 e	0.31 ± 0.10 a	11.63 ± 2.12 b	339.71 ± 68.83 c
VC	3.22 ± 0.42 abc	0.19 ± 0.04 abc	2.83 ± 0.36 bcd	24.05 ± 4.37 bc	25.13 ± 2.60 a	13.20 ± 2.12 cd	24.09 ± 2.71 c	n.d	23.50 ± 3.44 c	156.08 ± 24.23 b
OW	1.18 ± 0.39 a	0.09 ± 0.04 abc	3.89 ± 0.72 cd	16.04 ± 3.42 abc	111.34 ± 14.22 b	9.71 ± 1.91 bcd	2.44 ± 0.47 a	n.d	20.40 ± 3.52 c	48.77 ± 9.04 a
OL	2.40 ± 0.73 ab	0.06 ± 0.04 ab	4.96 ± 1.02 d	16.29 ± 3.35 abc	147.08 ± 26.73 b	12.27 ± 2.44 cd	3.54 ± 0.84 a	0.27 ± 0.06 a	25.10 ± 4.51 c	62.86 ± 11.38 a
GW	4.20 ± 1.74 bc	0.92 ± 0.85 c	2.67 ± 1.32 bcd	40.00 ± 16.23 cd	365.35 ± 38.10 d	12.41 ± 4.98 cd	10.33 ± 0.47 b	n.d	12.49 ± 5.49 b	190.05 ± 13.55 b

IO—Dry sludge rich in iron oxyhydroxides, MS—Dry marble sludge, CW—Carbonated waste of a peat exploitation, GS—Gypsum mining spoil, WS—Composted sewage sludge, BM—Bio-stabilised material of municipal solid wastes, VC—Vermicompost from pruning and gardening, OW—Composted solid olive-mill by-product irrigated with drinking water, OL—Composted solid olive-mill by-product irrigated with leachates of the olive-mill, GW—Composted greenhouse plant waste. Letters represent significant differences among waste materials (Kruskal–Wallis and Dunn tests, *p* < 0.05).

The artificial AMD prepared by oxidation of the Aznalcóllar’s toxic tailings discharged in the accident showed both the typical ultra-acid character (pH_(L)− _2.89 ± 0.03) and the extremely high EC_(L)_ (3.76 ± 0.14 dS m^−1^). Moreover, most of the PTE were present in high concentrations in AMD (Table 3). Below 100 µg L^−1^ were Ba, Be, In, Mo, Sc, Th, Tl, U, V, and Y; between 100 and 500 µg L^−1^ were Bi, Cd, Co, Cr, Ni, and Sn; between 500 and 1000 µg L^−1^ were Pb and Sb; and above 1000 µg L^−1^ were As, Cu, Mn, and Zn.

**Table 3:** Potentially toxic elements (PTE) concentration (mean ± st. dev) expressed in µg L^-1^ in the acid mine drainage (AMD) obtained from the oxidation of pyrite tailings of Aznalcóllar compared to regulatory levels.

PTE	AMD concentration (µg L^-1^)	Maximum level natural water by Spanish legislation^1^	Maximum admissible concentration in reclaimed water for irrigation by Spanish legislation^2^	Maximum admissible concentration in reclaimed water for irrigation by USA legislation^3^
As	**2859.70 ± 270.16**	50	100	100
Ba	34.27 ± 9.83	–	–	–
Be	3.19 ± 0.36	–	100	100
Bi	180.20 ± 10.98	–		–
Cd	**452.02 ± 5.82**	–	10	10
Co	**434.61 ± 8.20**	–	50	50
Cr	**351.89 ± 5.66**	50	100	100
Cu	**6238.22 ± 67.11**	120	200	200
In	31.56 ± 0.38	–	–	–
Mn	**12,937.64 ± 216.04**	–	200	200
Mo	6.33 ± 0.30	–	10	10
Ni	197.82 ± 1.89	–	200	200
Pb	597.69 ± 81.54	–	–	5000
Sb	817.85 ± 32.29	–	–	–
Sc	29.93 ± 0.19	–	–	–
Sn	443.91 ± 32.71	–	–	–
Th	8.25 ± 1.43	–	–	–
Tl	25.00 ± 2.19	–	–	
U	14.55 ± 0.68	–		–
V	54.92 ± 9.45	–	100	100
Y	39.86 ± 0.29	–	–	–
Zn	**32,208.45 ± 495.51**	500	–	2000

^1^“Normas de calidad ambiental (NCA)”: Concentration of a chemical in water, sediment, or biota, which must not be exceeded to protect human health and the environment set by Spanish legislation in Annex V of RD 817/2015^46^.

^2^Maximum admissible value (VMA): The highest level of a pollutant that is allowed in reclaimed water used for irrigation established by the Spanish legislation^47^.

^3^Recommended water quality criteria for irrigation: The highest level of a pollutant that is allowed in reclaimed water used for irrigation established by the United States Environmental Protection Agency^48^.

PTE that exceed regulatory levels in bold,

### Acid mine drainage treatment using waste materials

**Figure 2:**
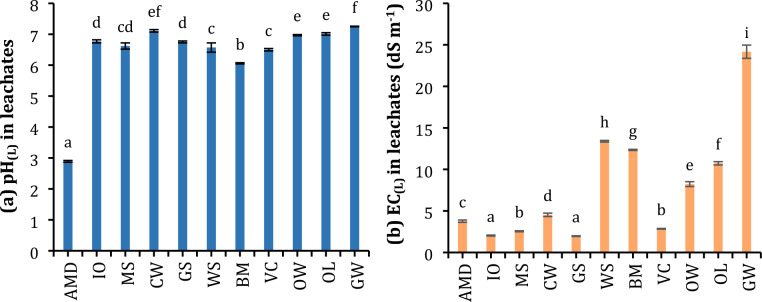
Variation of pH_(L)_ and EC_(L)_ in leachates resulting after the treatment of acid mine drainage (AMD) by the different waste materials. IO – Dry sludge rich in iron oxyhydroxides, MS – Dry marble sludge, CW – Carbonated waste of a peat exploitation, GS – Gypsum mining spoil, WS – Composted sewage sludge, BM – Bio-stabilised material of municipal solid wastes, VC – Vermicompost from pruning and gardening, OW – Composted solid olive-mill by-product irrigated with drinking water, OL – Composted solid olive-mill by-product irrigated with leachates of the olive-mill, GW – Composted greenhouse plant waste. Letters represent significant differences among waste materials (Kruskal–Wallis and Dunn tests, *p* < 0.05).

All leachates obtained after waste treatment showed a pH_(L)_ close to slightly acidic–neutral values (6–7.25), although with statistically significant differences among wastes (Fig. 2a). Whereas changes in EC_(L)_ due to waste treatment were quite heterogenous among the waste material used (EC_(L)_: 2–24 dS m^−1^). Some of them (IO, MS, GS, and VC) reduced the EC_(L)_ of the AMD; however, other wastes cause a significant increase in EC_(L)_ (GW, WS, BM, OW, and OL) between 2- and sixfold the EC measured in the AMD (Fig. 2b). Most PTE concentrations in the soluble fraction decreased significantly after waste treatments, although with large differences in removal effectiveness between organic and inorganic wastes (Table S2). Inorganic wastes showed a higher removal effectiveness of PTE than organic wastes, excluding VC which had similar removal rates to inorganic ones (Table 4). For the main PTE (As, Cd, Cr, Cu, Pb, Sb, and Zn), the retention rate of all tested inorganic wastes (IO, MS, CW, GS) as well as VC was above 95% in most cases and close to 100% for many of them. Thus, reducing the concentration of these elements to values below the regulatory levels in most cases. Similarly, the retention rate of other uncommon PTE such as In, Sc, Sn, Th, Tl, V and Y had also been outstanding, almost 100% in all inorganic wastes and VC. Furthermore, there were other less significant PTE for which the variability in retention rate is very high, such as Ba, Be, Bi, Co, Mn, Mo, Ni, and U. Among the inorganic wastes, dry sludge rich in iron oxyhydroxides (IO) had the highest capacity to retain PTE, followed by wastes with a high calcium carbonate content (MS: dry marble sludge, CW: carbonated waste from a peat exploitation). The gypsum spoil (GS) was not effective for Ba, Co, Mn, Mo, and Ni retention, but for other PTE it was as affective as the other inorganic wastes. On the other hand, most organic wastes demonstrated an overall good removal effectivity for these PTE, although lower than for inorganic wastes with the exception of VC. The wastes with lowest retention capacity for most PTE were BM and GW.

**Table 4:** Retention effectiveness of potentially toxic elements (PTE) of the inorganic and organic wastes expressed in %.

PTE	Waste materials									
	IO	MS	CW	GS	WS	BM	VC	OW	OL	GW
As	**99.96 ± 0.01 e**	**99.53 ± 0.27 e**	**99.74 ± 0.07 e**	**99.87 ± 0.04 e**	*69.56* ± *6.98 b*	***46.95* ± *3.49 a***	**99.67 ± 0.01 e**	*88.13* ± *3.68 d*	*81.72* ± *3.04 cd*	*77.20* ± *2.92 bc*
Ba	–	***40.27* ± *4.17 b***	***33.81* ± *36.49 ab***	***0.09* ± *9.27 a***	–	–	–	–	–	–
Be	**100.00 ± 0.00 e**	**99.95 ± 0.09 e**	**100.00 ± 0.00 e**	**99.63 ± 0.18 e**	*90.80* ± *2.04 d*	*70.53* ± *2.32 b*	**99.74 ± 0.45 e**	*80.77* ± *4.47 c*	*62.80* ± *4.60 a*	*80.46* ± *1.92 c*
Bi	**99.55 ± 0.24 d**	**96.82 ± 1.43 cd**	**97.93 ± 0.66 d**	**98.68 ± 0.18 d**	*80.87* ± *8.10 b*	*70.54* ± *2.20 a*	**99.76 ± 0.05 d**	*88.79* ± *2.27 cd*	*92.16* ± *0.14 cd*	**97.33 ± 0.49 d**
Cd	**98.91 ± 0.04 ef**	**99.28 ± 0.02 ef**	**99.68 ± 0.02 f.**	**95.82 ± 0.51 c**	**97.44 ± 0.30 d**	*75.12* ± *0.73 a*	**98.54 ± 0.07 df**	**98.26 ± 0.29 de**	*94.85* ± *0.30 c*	*89.65* ± *0.72 b*
Co	**98.80 ± 0.02 f.**	*64.25* ± *0.69 b*	**95.49 ± 0.22 f.**	***38.20* ± *6.54 a***	*83.57* ± *0.84 cd*	***39.48* ± *2.45 a***	**95.17 ± 0.59 f.**	*93.35* ± *0.33 ef*	*86.96* ± *0.47 de*	*79.95* ± *2.37 c*
Cr	**100.00 ± 0.00 b**	**100.00 ± 0.00 b**	**100.00 ± 0.00 b**	**100.00 ± 0.00 b**	*83.52* ± *2.65 b*	***16.57* ± *4.36 a***	**100.00 ± 0.00 b**	*87.72* ± *3.60 b*	*85.88* ± *1.63 b*	–
Cu	**99.93 ± 0.01 e**	**99.46 ± 0.03 e**	**98.45 ± 0.01 e**	**99.56 ± 0.05 e**	*92.49* ± *0.38 c*	*67.12* ± *0.90 a*	**99.82 ± 0.01 e**	**95.73 ± 0.48 d**	*90.04* ± *0.60 c*	*78.79* ± *2.42 b*
In	**100.00 ± 0.00 d**	**99.94 ± 0.07 d**	**100.00 ± 0.00 d**	**100.00 ± 0.00 d**	*91.86* ± *1.65 ab*	*91.39* ± *0.31 a*	**100.00 ± 0.00 d**	*94.38* ± *1.61 c*	*93.81* ± *0.58 bc*	*92.73* ± *0.84 abc*
Mn	**98.02 ± 0.16 b**	*78.43* ± *0.30 a*	*94.28* ± *0.30 b*	–	*73.12* ± *11.73 a*	–	*71.64* ± *2.50 a*	*75.19* ± *0.67 a*	*70.43* ± *1.03 a*	*69.54* ± *2.83 a*
Mo	***3.45* ± *81.16 a***	*73.25* ± *2.85 b*	–	–	–	–	–	–	–	–
Ni	*85.30* ± *0.16 e*	*62.98* ± *0.76 c*	*74.74* ± *0.54 d*	–	***19.98* ± *17.38 a***	–	*87.42* ± *1.24 e*	*69.88* ± *0.94 cd*	***47.79* ± *1.65 b***	***47.76* ± *6.51 b***
Pb	**100.00 ± 0.00 c**	**99.97 ± 0.05 c**	**100.00 ± 0.00 c**	**100.00 ± 0.00 c**	*93.19* ± *4.65 b*	*82.18* ± *1.30 a*	**99.93 ± 0.01 c**	*94.51* ± *1.83 b*	*90.90* ± *1.46 b*	*91.41* ± *1.38 b*
Sb	**99.33 ± 0.03 d**	**98.55 ± 0.06 d**	**98.13 ± 0.10 d**	**98.27 ± 0.14 d**	*62.07* ± *6.33 b*	***40.90* ± *3.51 a***	**98.27 ± 0.12 d**	*85.24* ± *2.59 c*	*84.31* ± *0.30 c*	*85.30* ± *1.91 c*
Sc	*91.83* ± *0.09 d*	**97.35 ± 0.43 d**	**95.55 ± 0.13 d**	**95.68 ± 0.21 d**	*77.58* ± *4.79 c*	*50.15* ± *2.12 a*	*90.34* ± *0.76 d*	*67.70* ± *5.87 b*	*71.76* ± *1.01 bc*	*78.65* ± *2.47 c*
Sn	**100.00 ± 0.00 c**	**99.38 ± 0.54 c**	**100.00 ± 0.00 c**	**100.00 ± 0.00 c**	***43.34* ± *22.57 a***	–	**99.70 ± 0.42 c**	*83.22* ± *4.71 b*	*87.11* ± *0.91 b*	*90.10* ± *2.49 b*
Th	**98.95 ± 0.51 d**	**95.88 ± 0.92 d**	**95.51 ± 1.03 d**	**97.33 ± 0.28 d**	*61.90* ± *8.95 c*	***16.58* ± *2.04 a***	**99.03 ± 0.21 d**	*56.40* ± *7.41 c*	*51.68* ± *1.85 bc*	43.92 ± 5.20 b
Tl	**95.86 ± 0.03 f.**	*60.10* ± *0.77 a*	*91.47* ± *0.16 ef*	*85.72* ± *1.47 c*	*90.98* ± *2.91 de*	*75.13* ± *0.68 b*	*91.70* ± *0.02 ef*	*86.42* ± *0.28 cd*	*83.24* ± *0.72 c*	*78.07* ± *3.96 b*
U	*80.20* ± *1.00 b*	*94.28* ± *0.17 c*	–	*91.96* ± *1.02 c*	*66.31* ± *7.12 a*	*79.39* ± *0.94 b*	*94.28* ± *1.37 c*	*74.45* ± *1.54 ab*	*71.68* ± *0.74 a*	–
V	**100.00 ± 0.00 b**	**100.00 ± 0.00 b**	**100.00 ± 0.00 b**	**100.00 ± 0.00 b**	–	–	**100.00 ± 0.00 b**	***22.11* ± *20.57 a***	–	–
Y	**99.93 ± 0.11 d**	**99.92 ± 0.06 d**	**99.87 ± 0.22 d**	**99.83 ± 0.08 d**	*79.26* ± *10.56 c*	***34.75* ± *2.33 a***	**99.67 ± 0.06 d**	*81.85* ± *3.88 c*	*59.57* ± *2.14 b*	*71.51* ± *0.99 c*
Zn	**99.22 ± 0.05 ef**	*93.63* ± *0.13 cd*	**99.86 ± 0.01 f.**	*92.87* ± *0.64 bc*	**95.17 ± 0.83 d**	*64.01* ± *1.57 a*	**97.92 ± 0.19 e**	**97.96 ± 0.39 ef**	**95.09 ± 0.36 d**	*91.09* ± *0.74 b*

IO—Dry sludge rich in iron oxyhydroxides, MS—Dry marble sludge, CW—Carbonated waste of a peat exploitation, GS—Gypsum mining spoil, WS—Composted sewage sludge, BM—Bio-stabilised material of municipal solid wastes, VC—Vermicompost from pruning and gardening, OW—Composted solid olive-mill by-product irrigated with drinking water, OL—Composted solid olive-mill by-product irrigated with leachates of the olive-mill, GW—Composted greenhouse plant waste. Letters represent significant differences among different waste materials for a same element (Kruskal–Wallis and Dunn tests, *p* < 0.05). Very high retention > 95% (bold), High retention > 50% (italic), Low retention < 50% (bold italic), No retention (–).

## Discussion

Physical, chemical, and biological characteristics of the waste materials reflect considerable differences in their composition. There are wastes with a strong carbonate character (CW and MS), others that are highly organic (VC, GW, OL, WS, OW, and BM), and also waste with high iron oxyhydroxide content (IO). These characteristics are selected by their important role in the immobilisation of PTE and the acid neutralisation^49–51^. For example, organic matter has a high affinity for some PTE because of the presence of ligands or functional groups^52^, in this order: Cu^2+^  > Hg^2+^  > Cd^2+^  > Fe^2+^  > Pb^2+^  > Ni^2+^  > Co^2+^  > Mn^2+^  > Zn^2+^  > As^5+^  > As^3+^^53,54^. Thus, organic matter together with total humic extract and humic and fulvic acids provide an important content of reactive colloidal fractions that allow the complexation of the different chemical forms of PTE^55,56^. Carbonates also exert a strong control over pH, which is considered a key property in controlling the immobilisation of most PTE because of its influence on the electrical charge of colloidal components^57^. In addition, it is a key component to neutralise acid solutions^40^. Likewise, iron oxyhydroxides content is another constituent to consider for the retention of some PTE, especially As, for which they exert a strong control on speciation and bioavailability^58,59^. In fact, the results of AMD treatment test indicate that many of the wastes tested show considerable acid neutralisation and PTE immobilisation capacity.

The concentration of most PTE in AMD was very high, exceeding the guideline values established by different legislations for As, Cd, Co, Cr, Cu, Mn, and Zn: (i) environmental quality standard for surface water in Spain^46^; (ii) legal regime for the reuse of treated water for irrigation in Spain^47^ and (iii) guidelines for water reuse in the USA^48^. The highest concentrations were found for As, Cd, Cu, and Zn, exceeding about 29-, 45-, 31-, and 16-fold the guideline values for reuse of treated water for irrigation according to Spanish legislation and United States Environmental Protection Agency (US EPA)^47,48^; besides, other PTE such as Co, Cr, Cu, and Mn, were also considered relevant in relation to their high concentrations also exceeding these regulatory levels. Other elements like Pb, Sb, and Tl presented potentially concerning concentrations, although their guideline values are not included in the previous references. In addition, most of the PTE in this acid mine drainage were at much higher concentrations than those found in the acidic water discharged in the Aznalcóllar mine accident^42^, as well as the concentrations in AMD generated in metal mines in Australia^60^ or in other mining areas around the world^8^. Thus, the results in this study can be extrapolated to most acid mine waters treatment situations around the world; moreover, the use of the wastes tested in this study to treat real AMD worldwide would most likely produce a better quality treated water than that achieved for the artificial AMD used in this study.

The treatment of AMD with wastes has been effective in neutralising the acidity in all cases. The pH in treated water increases from pH < 3 to values above 6 and close to neutrality depending on the waste used. In this sense, although the role of carbonates in the neutralisation of acid mine drainage has already been widely demonstrated^61,62^, no statistical correlation was found between pH in leachate (pH_(L)_) and the CaCO_3_ concentration in the different wastes (Table S3). Nevertheless, carbonates are not the only buffering components controlling pH; there are other constituents in the wastes (e.g., organic matter, exchange bases, Fe and Al oxides, silicates) with relevant influence in this process capacity^63,64^. Likewise, the concentrations of several PTE in AMD after treatment with wastes have been significantly reduced. Indeed, the removal efficiencies of PTE obtained with these wastes have been much higher than those achieved in other studies^8,9,65^. Among the wastes used, inorganic wastes were much more effective in retaining PTE than organic ones. The decreasing order of effectiveness was as follows: IO > CW ≥ MS ≥ VC > GS > OW > OL > WS > GW > BM; where wastes rich in iron oxyhydroxides and carbonates are more effective in the retention of PTE than wastes rich in organic matter. The removal rates for wastes dominated by carbonates (CW and MS) or iron oxyhydroxides (IO) are above 95% for most PTE present in AMD, while for organic wastes the removal rate was below 95% in most cases, with values as low as 15% in the case of bio-stabilised material of municipal solid wastes (BM). In other studies, for similar wastes the removal rates achieved were similar or even lower. For example, water filters partly made of iron-rich materials achieved removal rates of 50% for As^66^. However, other studies that also explore As retention capacity of water filters with iron oxide-rich materials reached rates of 90%^67^ and 99%^68^. The latter study concerned not only filters made from iron-rich waste, but also marble slurry filters for which As removal rate is 95%^68^. Furthermore, the success of these materials is not limited to As; for example, along with near 100% As retention in groundwater affected by an abandoned gold mine when treated with various mixtures composed of organic carbon, zero-valent iron and limestone, a strong decrease in the concentration of Al, Cd, Co, Cu and Ni has been demonstrated^69^; although the concentrations of these elements in the groundwaters are much lower than in our study. On the other hand, although less studied, the capacity of some organic wastes has also been assessed; for example, it has been reported a 70% reduction of some PTE (Al, As, Cd, Cu, Fe, Ni, Mn, Pb, and Zn) present in sulfide mine leachates by the addition of aqueous organic wastes from domestic wastewater^16^. Agricultural wastes have also been used to remove pollutants; for example, solid-olive mill by-products have a great capacity to remove Cr, Mn, Cu, Zn, Ni, and Pb from mining wastewater^70^. Similarly, there is an extensive list of agricultural waste (agave, bananas, wheat, rice, citrus fruits) that have been used for the immobilisation of different PTE (Cd, Pb, Zn) with uncertain results^71^. Particularly noteworthy is the case of vermicompost (VC), which shows retention rates of PTE close to those of carbonated and iron-rich wastes. This may be due to the higher content of calcium carbonate and total iron compared to other organic wastes, and, to a lesser extent, its considerable high OC content. In this sense, vermicompost can be a very effective material for the treatment of AMD. A similar study for the treatment of AMD^72^ using vermicompost and other agricultural by-products (sheep, cow, and rabbit manure) reported retention rates of 90% for As, Cd, Cu, and Zn in AMD. Similarly, gypsum spoil (GS) also has a high retention capacity for PTE similar to that of the other inorganic wastes, although for some, such as Ni and Co, was very low. The high retention capacity of GS is related to high CaCO_3_ and FeT contents.

Equally, it should not be overlooked that the content of PTE in some wastes may pose a potential risk. In relation to the initial concentration of PTE in the wastes, sludge rich in iron oxyhydroxide and gypsum spoil presented slightly high concentrations of As, Pb and Sb. However, they do not exceed the guideline values to declare a soil polluted according to the regional regulations^73^ or the maximum levels that a compound must have in order to be used as a fertiliser product in Spain^74^. The rest of the inorganic wastes have low concentrations of most PTE. The same applies to organic wastes, although some of them show high concentrations of certain PTE (Cr, Cu, V and Zn), they do not exceed the guideline values. In particular, the organic wastes with the highest concentrations are compost from greenhouse waste (GW), composted sewage sludge (WS), and bio-stabilised material from municipal solid waste treatment (BM); which are also the wastes with lowest retention capacity. The presence of PTE in waste related to urban activities is common^75,76^, although in our case they do not exceed the guidelines values and, therefore, pose a low risk of PTE pollution. Anyway, concern should be raised about their use due to the very high salinity reflected in their high EC values. In fact, most of the organic wastes except vermicompost cause an increase in EC in the leachates resulting from the treatment with respect to AMD.

The main PTE (As, Cd, Cr, Cu, Pb, and Zn) have been successfully removed (close to 100%) from AMD by waste treatment. Especially inorganic wastes and vermicompost have the highest capacity, leaving the concentrations of most of them in the treated water below the regulatory levels for irrigation and surface water in Spain^46–48^. In contrast, in the treatment with the organic wastes, although significantly reduced the PTE concentrations, the values were above the regulatory levels in most cases. However, the retention of other less studied PTE such as In, Sc, Sn, Th, Tl, V and Y is also remarkable. Promising results are obtained for specific elements, as in the case of V, where previous studies with commercial iron products and a ferric residue from groundwater treatment obtained 85% of removal of this element from mining water^77^, compared to values close to 100% removal in our study for inorganic and vermicompost wastes. Thallium is another highly toxic element and quite understudied^78^; and the treatment and removal in wastewater is one of the major challenges in the coming years^79^. In our study, the removal rate of Tl in AMD is above 75% for all wastes analysed and for some wastes such as IO, CW, WS and VC above 90%, whereas in other studies included in^78^, the reduction of Tl in wastewaters after treatment with lime is between 21 and 49%. Antimony is also considered a concern element due to the potential toxicity in surface and groundwater; and the use of commercial coagulants such as iron salts have proven to be effective in remediating Sb-polluted waters; in this case, the ferric chloride coagulant presented removal rates higher than 80% across a broad pH range^80^. The efficiency of Sb removal from AMD in our study is higher than 95% for inorganic and vermicompost wastes, which shows the high potential application of the wastes that we have analysed.

Nowadays, the demand of many elements is projected to be high to achieve the energy transition and mining is an essential activity which is reactivating. The production and availability of technology-critical elements is also a current concern. In this scenario, the potential pollution and widespread of PTE into the environment is predicted to rise in the short-term, together with the production of waste related to the different human activities. This study is in line with both problems (increased input of pollutants into the environment and increased production of waste), so the promising results obtained may contribute to the environmental protection and human safety.

## Conclusions

This study tests the effectiveness of various wastes as a potential treatment of acid mine drainage to promote mine restoration and environmental protection by the sustainable management of urban, mining, and agro-industrial wastes in a circular economy scenario. Our results conclude that the waste materials studied have a very high acid neutralising capacity, as well as a strong capacity to retain potentially toxic elements. Inorganic wastes, together with vermicompost from pruning and gardening, reduced by more than 95% the concentrations of most PTE in a highly polluted simulated AMD, while organic wastes retain between 50 and 95%. The potential effectiveness followed this order: IO > CW ≥ MS ≥ VC > GS > OW > OL > WS > GW > BM. Thus, a wide range of mining, urban, and agro-industrial wastes could be recovered for use in the treatment of AMD. The use of these wastes as AMD treatment technique showed promising results to be applied in the decontamination of polluted waters and as a control technique on tailing deposits to prevent the AMD generation. This study is the first step in the development of green technologies based on the different combinations of wastes with contrasting characteristics, to create solution (e.g.: Technosols, permeable reactive barriers, etc.) with a higher capacity to retain a greater variety of PTE and reduce acidity in polluted environments. The use of waste to remediate AMD will decrease the cost of the water treatment. This is especially relevant for the rehabilitation of areas with historical or abandoned mines, where the decrease in cost by replacing commonly used and expensive reagents for worthless waste will increase the affordability of water treatments. Nevertheless, additional site-specific studies should be conducted to include the cost of waste transport, as well as to evaluate the *in-situ* effectiveness of waste combinations under real field conditions.

## Supplementary Information

Supplementary Information.

## Data Availability

The authors confirm that the data supporting the findings of this study are available within the article and its Supplementary materials. Moreover, the datasets used and/or analysed during the current study are available from the corresponding author on reasonable request.
