# Supplementary material for: Remediation potential of mining, agro-industrial, and urban wastes against acid mine drainage
Source: Sci Rep. 2023 Jul 26;13:12120. doi: 10.1038/s41598-023-39266-4 (PMC10372011; doi:10.1038/s41598-023-39266-4)
Supplement: Supplementary file 1 — Supplementary Information. [file 41598_2023_39266_MOESM1_ESM.docx]

**Supplementary material**


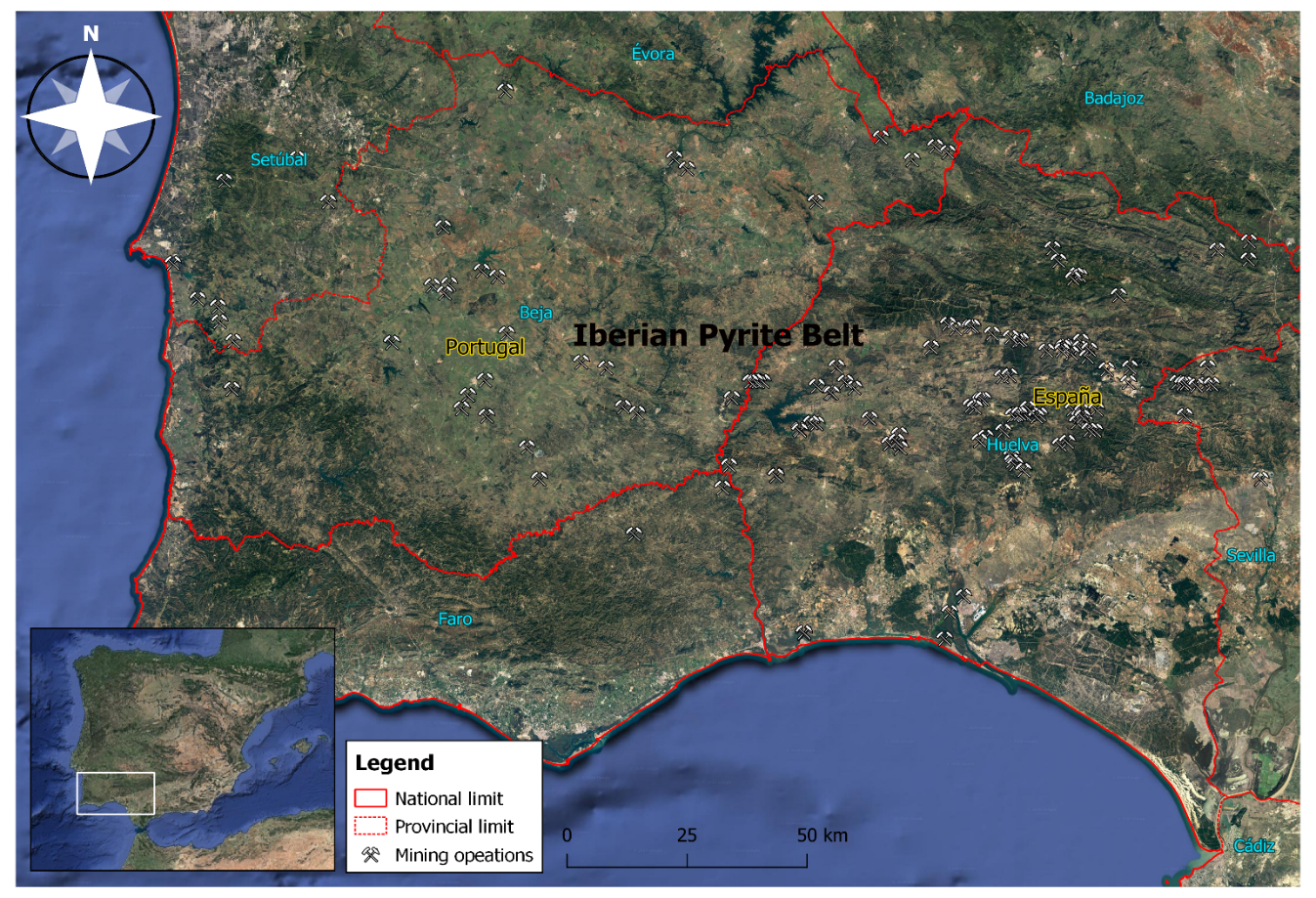


**Fig. S1.** Location of the Iberian Pyrite Belt and massive sulfides mining operations covering the districts of Beja, Setúbal and Faro in Portugal, and the provinces of Huelva and Sevilla in Spain. This satellite imagery was generated using QGIS 3.20 Odense software (<https://www.qgis.org/es/site/index.html>), orthophotography provided by OpenStreetMap (<https://www.openstreetmap.org/#map=6/40.007/-2.488>) and environmental information on mining locations by REDIAM – Andalusian Environmental Information Network (Spain) (<https://portalrediam.cica.es/descargas>).

**Table S1.**

Potentially toxic elements (PTE) content of the toxic tailing used to obtain the artificial acid mine drainage (AMD) expressed in mg kg^-1^.

| PTE | Maximum | Minimum | Median |
| --- | --- | --- | --- |
| Ag | 41.0 | 25.0 | 34.0 ± 4.8 * |
| As | 4122.6 | 1473.0 | 3749.0 |
| Ba | 804.1 | 353.7 | 650.8 |
| Be | 1.7 | 0.4 | 0.7 |
| Bi | 86.6 | 37.9 | 76.4 |
| Cd | 36.8 | 22.9 | 28.6 |
| Ce | 50.0 | 13.0 | 28.0 ± 9.1 * |
| Co | 54.0 | 32.6 | 49.8 |
| Cr | 78.6 | 34.5 | 63.4 |
| Cu | 2175.8 | 1100.7 | 2009.1 |
| Hg | 4.9 | 2.4 | 3.2 |
| In | 3.0 | 1.5 | 2.7 |
| La | 40.0 | 12.0 | 21.0 ± 7.2 * |
| Mn | 1097.5 | 667.3 | 726.3 |
| Mo | 8.6 | 3.2 | 7.8 |
| Ni | 31.4 | 15.8 | 18.7 |
| Pb | 9635.9 | 4352.9 | 9507.3 |
| Sb | 957.9 | 394.1 | 786.2 |
| Sc | 7.1 | 0.0 | 3.0 |
| Se | 5.3 | 0.0 | 4.4 |
| Sn | 24.0 | 6.0 | 7.3 |
| Tl | 66.8 | 33.1 | 57.1 |
| Th | 6.9 | 3.3 | 4.6 |
| U | 2.4 | 1.5 | 2.2 |
| V | 78.0 | 34.8 | 35.9 |
| Y | 14.4 | 5.2 | 6.5 |
| Zn | 8063.4 | 6246.6 | 7183.5 |

Data belonging to ^42^, except those with asterisks that belong to ^44^. Those with asterisks are mean values, not median.

**Table S2**

Potentially toxic elements (PTE) concentration (mean ± st. dev) expressed in µg L^-1^ in leachates resulting from the treatment of the artificial acid mine drainage (AMD) by the different waste materials.

|  |  | **Waste materials** | | | | | | | | | |
| --- | --- | --- | --- | --- | --- | --- | --- | --- | --- | --- | --- |
| **PTE**  **(µg L^-1^)** | **AMD** | **IO** | **MS** | **CW** | **GS** | **WS** | **BM** | **VC** | **OW** | **OL** | **GW** |
| **As** | 2859.70 ± 270.16 e | 1.16 ± 0.13 a | 13.51 ± 7.85 a | 7.48 ± 1.99 a | 3.84 ± 0.96 a | 870.45 ± 199.50 c | 1517.10 ± 99.78 d | 9.57 ± 0.47 a | 339.45 ± 105.17 ba | 522.84 ± 86.88 b | 652.12 ± 83.41 bc |
| **Ba** | 34.27 ± 9.83 ba | 74.91 ± 1.67 bc | 20.47 ± 1.43 a | 22.69 ± 12.51 a | 34.24 ± 3.18 ba | 103.20 ± 37.49 cd | 128.26 ± 15.69 de | 162.25 ± 8.46 e | 652.16 ± 20.47 g | 794.77 ± 25.55 h | 401.61 ± 14.63 f |
| **Be** | 3.19 ± 0.36 e | 0.00 ± 0.00 a | 0.00 ± 0.00 a | 0.00 ± 0.00 a | 0.02 ± 0.01 a | 0.29 ± 0.07 ab | 0.94 ± 0.07 cd | 0.01 ± 0.02 a | 0.62 ± 0.15 bc | 1.19 ± 0.15 d | 0.62 ± 0.06 bc |
| **Bi** | 180.20 ± 10.98 e | 0.82 ± 0.43 a | 5.72 ± 2.56 ab | 3.74 ± 1.18 ab | 2.37 ± 0.33 a | 34.48 ± 14.60 c | 53.09 ± 3.96 d | 0.44 ± 0.08 a | 20.21 ± 4.08 bc | 14.12 ± 0.25 ab | 4.81 ± 0.87 ab |
| **Cd** | 452.02 ± 5.82 f | 4.94 ± 0.15 ab | 3.27 ± 0.10 a | 1.47 ± 0.06 a | 18.87 ± 2.29 c | 11.56 ± 1.32 b | 112.47 ± 3.30 e | 6.59 ± 0.31 ab | 7.86 ± 1.32 ab | 23.27 ± 1.32 c | 46.80 ± 3.27 d |
| **Co** | 434.61 ± 8.20 g | 5.23 ± 0.09 a | 155.37 ± 3.01 e | 19.59 ± 0.95 a | 268.59 ± 28.43 f | 71.38 ± 3.63 cd | 263.02 ± 10.66 f | 20.97 ± 2.56 a | 28.88 ± 1.41 ba | 56.66 ± 2.03 bc | 87.17 ± 10.31 d |
| **Cr** | 351.89 ± 5.66 bc | 0.00 ± 0.00 a | 0.00 ± 0.00 a | 0.00 ± 0.00 a | 0.00 ± 0.00 a | 58.01 ± 9.33 a | 293.58 ± 15.32 bc | 0.00 ± 0.00 a | 43.20 ± 12.69 a | 49.69 ± 5.72 a | 374.82 ± 86.08 c |
| **Cu** | 6238.22 ± 67.11 f | 4.47 ± 0.43 a | 33.56 ± 2.12 a | 96.91 ± 0.29 a | 27.72 ± 2.93 a | 468.53 ± 23.56 c | 2051.00 ± 56.47 e | 11.38 ± 0.35 a | 266.31 ± 29.56 b | 621.56 ± 37.37 c | 1323.50 ± 150.79 d |
| **In** | 31.56 ± 0.38 d | 0.00 ± 0.00 a | 0.02 ± 0.02 a | 0.00 ± 0.00 a | 0.00 ± 0.00 a | 2.57 ± 0.52 c | 2.72 ± 0.10 c | 0.00 ± 0.00 a | 1.78 ± 0.51 bc | 1.96 ± 0.19 bc | 2.29 ± 0.27 bc |
| **Mn** | 12937.64 ± 216.04 d | 256.76 ± 21.24 a | 2791.30 ± 38.72 bca | 740.01 ± 39.57 ba | 28157.19 ± 3067.08 e | 3476.60 ± 1517.82 bc | 15777.99 ± 582.29 d | 3668.79 ± 323.63 bc | 3209.11 ± 87.35 bca | 3825.76 ± 133.26 bc | 3940.75 ± 366.19 c |
| **Mo** | 6.33 ± 0.30 a | 6.11 ± 5.14 a | 1.70 ± 0.18 a | 424.28 ± 18.58 b | 8.05 ± 1.21 a | 138.45 ± 49.01 a | 174.72 ± 7.15 a | 12.31 ± 0.72 a | 14.35 ± 1.50 a | 14.00 ± 0.45 a | 1568.89 ± 222.68 c |
| **Ni** | 197.82 ± 1.89 de | 29.08 ± 0.32 ab | 73.24 ± 1.50 bc | 49.96 ± 1.06 abc | 220.00 ± 22.01 e | 158.30 ± 34.39 d | 848.79 ± 27.45 f | 24.90 ± 2.45 a | 59.58 ± 1.85 abc | 103.29 ± 3.26 c | 103.34 ± 12.87 c |
| **Pb** | 597.69 ± 81.54 c | 0.00 ± 0.00 a | 0.18 ± 0.32 a | 0.00 ± 0.00 a | 0.00 ± 0.00 a | 40.70 ± 27.76 ab | 106.51 ± 7.74 b | 0.42 ± 0.05 a | 32.85 ± 10.93 ab | 54.38 ± 8.73 ab | 51.36 ± 8.27 ab |
| **Sb** | 817.85 ± 32.29 e | 5.50 ± 0.27 a | 11.83 ± 0.47 a | 15.34 ± 0.81 a | 14.12 ± 1.13 a | 310.24 ± 51.76 c | 483.36 ± 28.70 d | 14.19 ± 0.93 a | 120.71 ± 21.16 b | 128.32 ± 2.43 b | 120.21 ± 15.61 b |
| **Sc** | 29.93 ± 0.19 e | 2.45 ± 0.03 a | 0.80 ± 0.13 a | 1.34 ± 0.04 a | 1.30 ± 0.06 a | 6.72 ± 1.44 b | 14.92 ± 0.64 d | 2.89 ± 0.23 a | 9.67 ± 1.76 c | 8.46 ± 0.30 bc | 6.39 ± 0.74 b |
| **Sn** | 443.91 ± 32.71 c | 0.00 ± 0.00 a | 2.75 ± 2.41 a | 0.00 ± 0.00 a | 0.00 ± 0.00 a | 251.51 ± 100.17 b | 1224.84 ± 55.40 d | 1.34 ± 1.86 a | 74.47 ± 20.92 a | 57.20 ± 4.02 a | 43.94 ± 11.04 a |
| **Th** | 8.25 ± 1.43 c | 0.09 ± 0.04 a | 0.34 ± 0.08 a | 0.37 ± 0.09 a | 0.22 ± 0.03 a | 3.15 ± 0.74 b | 6.89 ± 0.17 c | 0.08 ± 0.02 a | 3.60 ± 0.61 b | 3.99 ± 0.15 b | 4.63 ± 0.43 b |
| **Tl** | 25.00 ± 2.19 f | 1.04 ± 0.01 a | 9.98 ± 0.19 e | 2.14 ± 0.04 ab | 3.57 ± 0.37 bc | 2.26 ± 0.73 ab | 6.22 ± 0.17 d | 2.08 ± 0.01 ab | 3.40 ± 0.07 bc | 4.19 ± 0.18 bcd | 5.49 ± 0.99 cd |
| **U** | 14.55 ± 0.68 c | 2.89 ± 0.15 ba | 0.83 ± 0.02 a | 39.31 ± 3.82 d | 1.17 ± 0.15 ba | 4.90 ± 1.04 b | 3.00 ± 0.14 ba | 0.83 ± 0.20 a | 3.72 ± 0.23 ba | 4.12 ± 0.11 ba | 16.31 ± 1.95 c |
| **V** | 54.92 ± 9.45 bc | 0.00 ± 0.00 a | 0.00 ± 0.00 a | 0.00 ± 0.00 a | 0.00 ± 0.00 a | 85.29 ± 15.20 c | 139.09 ± 12.21 d | 0.00 ± 0.00 a | 42.78 ± 11.30 b | 72.57 ± 9.86 bc | 147.81 ± 28.19 d |
| **Y** | 39.86 ± 0.29 f | 0.03 ± 0.04 a | 0.04 ± 0.02 a | 0.05 ± 0.09 a | 0.07 ± 0.04 a | 8.27 ± 4.21 bc | 26.01 ± 0.93 e | 0.14 ± 0.02 a | 7.24 ± 1.55 bc | 16.11 ± 0.85 d | 11.36 ± 0.40 c |
| **Zn** | 32208.45 ± 495.51 e | 259.29 ± 14.41 a | 2050.18 ± 43.75 b | 45.44 ± 2.20 a | 2297.60 ± 207.18 bc | 1558.11 ± 267.60 b | 11592.10 ± 504.43 d | 669.57 ± 60.86 a | 655.22 ± 126.60 a | 1581.21 ± 114.97 b | 2869.79 ± 240.30 c |

IO – Dry sludge rich in iron oxyhydroxides, MS – Dry marble sludge, CW – Carbonated waste of a peat exploitation, GS – Gypsum mining spoil, WS – Composted sewage sludge, BM – Bio-stabilised material of municipal solid wastes, VC – Vermicompost from pruning and gardening, OW – Composted solid olive-mill by-product irrigated with drinking water, OL – Composted solid olive-mill by-product irrigated with leachates of the olive-mill, GW – Composted greenhouse plant waste. Letters represent significant differences among different waste materials for a same element (Kruskal-Wallis and Dunn tests, p < 0.05).

**Table S3.**

Correlation coefficients (Spearman) relating potentially toxic elements (PTE) concentrations in treated waters with waste properties (EC: electrical conductivity; OC: organic carbon content; CaCO_3_: calcium carbonate content; CEC: cation exchange capacity; N_T_/C_T_: total concentrations of N and C; BR: basal respiration rate). See Table 2 for waste properties and Table S1 for PTE concentration in the treated waters. Correlations ≥ 0.7 are black boldfaced.

|  | **pH** | **EC** | **OC** | **CEC** | **CaCO_3_** | **N_T_** | **C_T_** | **Fe_T_** | **P_A_** | **SR** |
| --- | --- | --- | --- | --- | --- | --- | --- | --- | --- | --- |
| **pH_(L)_** | **0.771**** | -0.042 | 0.109 | 0.227 | 0.175 | -0.031 | -0.017 | -0.280 | 0.473** | 0.480** |
| **EC_(L)_** | 0.156 | **0.725**** | 0.696** | **0.735**** | -0.595** | **0.814**** | **0.782**** | -0.238 | 0.179 | -0.133 |
| **As_(L)_** | -0.009 | **0.766**** | 0.601** | 0.672** | -0.582** | **0.809**** | **0.840**** | -0.170 | 0.001 | -0.311 |
| **Ba_(L)_** | 0.376* | 0.172 | 0.697** | **0.818**** | -0.390* | 0.646** | 0.644** | 0.232 | -0.048 | 0.208 |
| **Be_(L)_** | 0.220 | 0.648** | 0.501* | **0.794**** | -0.533** | **0.707**** | **0.865**** | 0.110 | 0.097 | -0.054 |
| **Bi_(L)_** | -0.063 | **0.733**** | 0.178 | 0.614** | -0.469** | **0.721**** | **0.799**** | -0.213 | -0.031 | -0.430* |
| **Cd_(L)_** | -0.008 | **0.715**** | 0.420* | 0.553** | **-0.712**** | 0.491** | 0.596** | 0.363* | 0.129 | -0.142 |
| **Co_(L)_** | -0.061 | 0.631** | -0.038 | -0.012 | -0.207 | 0.051 | 0.231 | -0.059 | 0.085 | -0.484** |
| **Cr_(L)_** | 0.110 | **0.774**** | 0.529** | 0.694** | **-0.723**** | **0.763**** | **0.758**** | 0.039 | 0.064 | -0.250 |
| **Cu_(L)_** | 0.179 | **0.831**** | 0.483** | 0.664** | -0.537** | **0.703**** | **0.853**** | -0.200 | 0.182 | -0.150 |
| **In_(L)_** | -0.005 | **0.750**** | 0.421* | 0.623** | -0.637** | **0.768**** | **0.780**** | -0.124 | -0.070 | -0.448** |
| **Mn_(L)_** | -0.025 | 0.539* | 0.285 | 0.265 | -0.282 | 0.198 | 0.340 | 0.260 | 0.273 | -0.026 |
| **Mo_(L)_** | 0.134 | 0.649* | 0.498** | 0.540** | -0.421* | 0.583** | 0.577** | -0.166 | 0.483** | 0.198 |
| **Ni_(L)_** | -0.182 | **0.831**** | -0.023 | 0.257 | -0.498** | 0.306 | 0.423* | 0.021 | 0.236 | -0.459** |
| **Pb_(L)_** | 0.012 | 0.675** | 0.614** | **0.743**** | -0.643** | **0.800**** | **0.852**** | 0.056 | -0.060 | -0.210 |
| **Sb_(L)_** | -0.087 | **0.843**** | 0.504** | **0.775**** | -0.608** | **0.857**** | **0.878**** | -0.061 | 0.249 | -0.138 |
| **Sc_(L)_** | -0.048 | 0.523** | 0.528** | **0.829**** | -0.677** | **0.809**** | **0.864**** | 0.293 | -0.092 | -0.020 |
| **Sn_(L)_** | -0.113 | 0.654** | 0.507** | **0.713**** | -0.596** | **0.856**** | **0.851**** | -0.051 | -0.149 | -0.399* |
| **Th_(L)_** | 0.211 | **0.782**** | 0.402* | 0.642** | -0.507** | 0.663** | **0.830**** | -0.154 | 0.102 | -0.173 |
| **Tl_(L)_** | 0.371* | 0.418* | 0.114 | 0.082 | 0.008 | 0.065 | 0.368* | -0.304 | -0.140 | -0.301 |
| **U_(L)_** | 0.337 | 0.450* | 0.260 | 0.515** | -0.264 | 0.472** | 0.434* | -0.338 | 0.542** | 0.214 |
| **V_(L)_** | 0.077 | **0.784**** | 0.535** | 0.695** | **-0.750**** | **0.755**** | **0.780**** | 0.032 | 0.065 | -0.245 |
| **Y_(L)_** | 0.046 | 0.665** | 0.596** | **0.779**** | -0.624** | **0.759**** | **0.865**** | 0.119 | 0.033 | -0.121 |
| **Zn_(L)_** | -0.008 | 0.622** | 0.157 | 0.082 | -0.398* | 0.134 | 0.301 | 0.098 | -0.023 | -0.407* |

* Significant correlation p < 0.05, ** Significant correlation p < 0.01
